# Supplementary material for: Quantum walk processes in quantum devices
Source: Heliyon. 2023 Feb 8;9(3):e13416. doi: 10.1016/j.heliyon.2023.e13416 (PMC9988498; doi:10.1016/j.heliyon.2023.e13416)
Supplement: MMC — Two tables of parameters, Table II and Table III for the quantum circuit used in Fig. 4(c) and Fig. 5(b) respectively. [file mmc1.pdf]

SUPPLEMENTAL INFORMATION

In this section we provide two tables of parameters, Table. II and Table. III for the quantum circuit used in Fig. 4(c) and Fig. 5(b) respectively.

All 4-qubit circuits were executed on the "ibmq_bogota" IBM quantum processor, and all 20-qubit circuits were executed on the "ibmq_paris" IBM quantum processor.

The code and data related to this work is provided in a public GitHub repository: https://github.com/q-ml/quantum-walks-quantum-devices

**Parameters**	**R_z Gates**																						
	1	2	3	4	5	6	7	8	9	10	11	12	13	14	15	16	17	18	19	20	21	22	23
θ	-1.57	-0.64	1.57	-0.64	0.79	4.18	-2.37	4.18	8.82	8.82	-1.86	-1.86	1.57	1.57	4.76	4.76	11.3	11.3	0.79	-1.05	0.79	-1.05	-3.14
	24	25	26	27	28	29	30	31	32	33	34	35	36	37	38	39	40	41	42				
θ	-1.76	-3.14	-1.76	2.09	2.09	-1.86	-1.86	1.57	1.57	4.76	4.76	11.3	11.3	-3.14	-1.57	-3.14	-1.57	0.76	-2.38				

TABLE II. The R_z gate parameters used in the circuit in Fig. 4(c) are given in the order from top to bottom, left to right.

**Parameters**	**R_z Gates**																						
	1	2	3	4	5	6	7	8	9	10	11	12	13	14	15	16	17	18	19	20	21	22	23
θ	-0.12	-0.49	4.59	4.32	11.74	11.15	-1.57	1.5	3.46	5.52	7.85	12.51	1.57	-2.56	2.85	4.2	10.07	2.32	2.02	4.59	5.32	12.44	7.75

TABLE III. The R_z gate parameters used in the circuit in Fig. 5(b) are given in the order from top to bottom, left to right.
